# Supplementary material for: Orchestrating chromosome conformation capture analysis with Bioconductor
Source: Nat Commun. 2024 Feb 5;15:1072. doi: 10.1038/s41467-024-44761-x (PMC10844600; doi:10.1038/s41467-024-44761-x)
Supplement: Supplementary file 1 — Supplementary information [file 41467_2024_44761_MOESM1_ESM.pdf]

Supplemental information

Orchestrating chromosome conformation capture analysis with Bioconductor

Jacques Serizay 1, *
Cyril Matthey-Doret 1,2†
Amaury Bignaud 1,2
Lyam Baudry 1,2‡
Romain Koszul 1

1: Institut Pasteur, CNRS UMR3525, Université Paris Cité, Unité Régulation Spatiale des Génomes, Paris, France

2: Sorbonne Université, Collège Doctoral, Paris, France

†: Present address: Swiss Data Science Center, École Polytechnique Fédérale de Lausanne, 1015 Lausanne, Switzerland

‡: Present address: Université de Lausanne, Center for Integrative Genomics, Quartier Sorge 1015

* Corresponding author: jacques.serizay@pasteur.fr

A

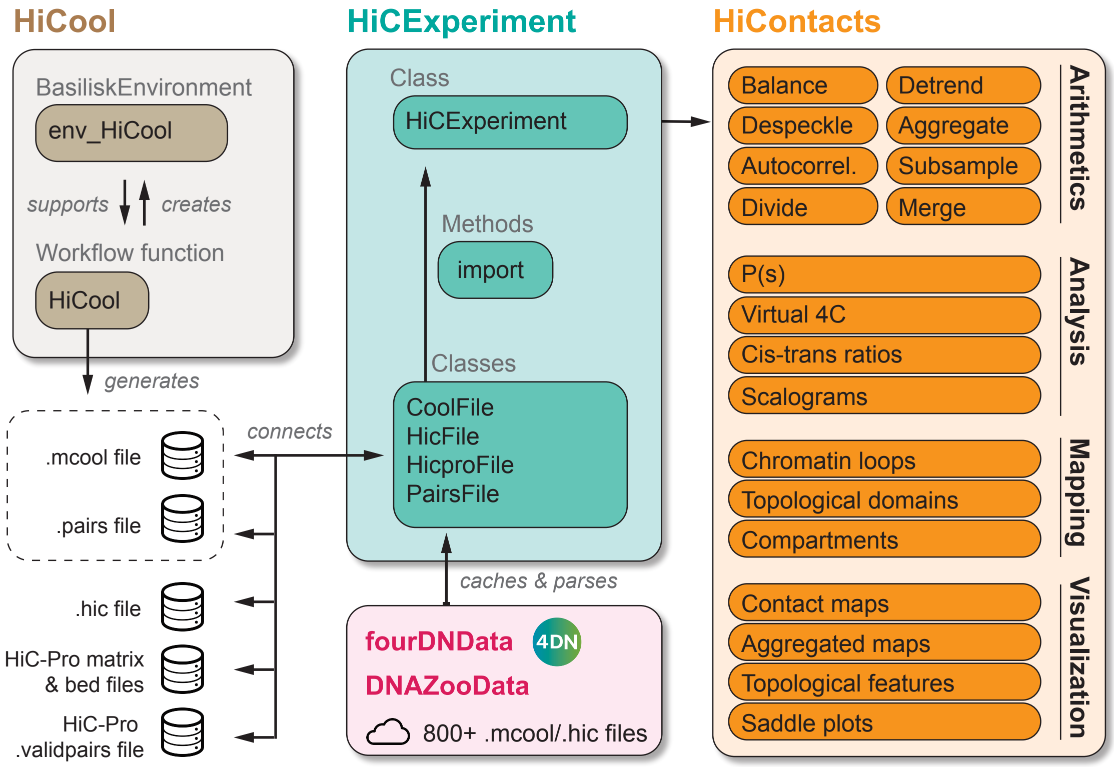

HiCool
BasiliskEnvironment
env_HiCool
supports
creates
Workflow function
HiCool
generates
.mcool file
.pairs file
.hic file
HiC-Pro matrix & bed files
HiC-Pro .validpairs file
connects
HiCExperiment
Class
HiCExperiment
Methods
import
Classes
CoolFile
HicFile
HicproFile
PairsFile
caches & parses
fourDNData
4DN
DNAZooData
800+ .mcool/.hic files
HiContacts
Balance
Detrend
Despeckle
Aggregate
Autocorrel.
Subsample
Divide
Merge
Arithmetics
P(s)
Virtual 4C
Cis-trans ratios
Scalograms
Analysis
Chromatin loops
Topological domains
Compartments
Mapping
Contact maps
Aggregated maps
Topological features
Saddle plots
Visualization

Supplemental figures and tables

Supplementary Figure 1: Ecosystem built on the *HiCExperiment* data structure
HiCool package automatically creates a self-managed conda environment to process raw Hi-C .fastq files within R and returns two pointers (*ContatFile* and *PairsFile*) to the disk-stored output pairs and binned matrix files. These connections can be parsed, allowing random access, in R using *import*. This instantiates a *HiCExperiment* object. *HiCExperiment* objects can also be directly created using two data gateway packages, *fourDNData* and *DNAZooData*. Visualization, arithmetic and in-depth analyses within and between *HiCExperiment* objects is possible thanks to the *HiContacts* package.

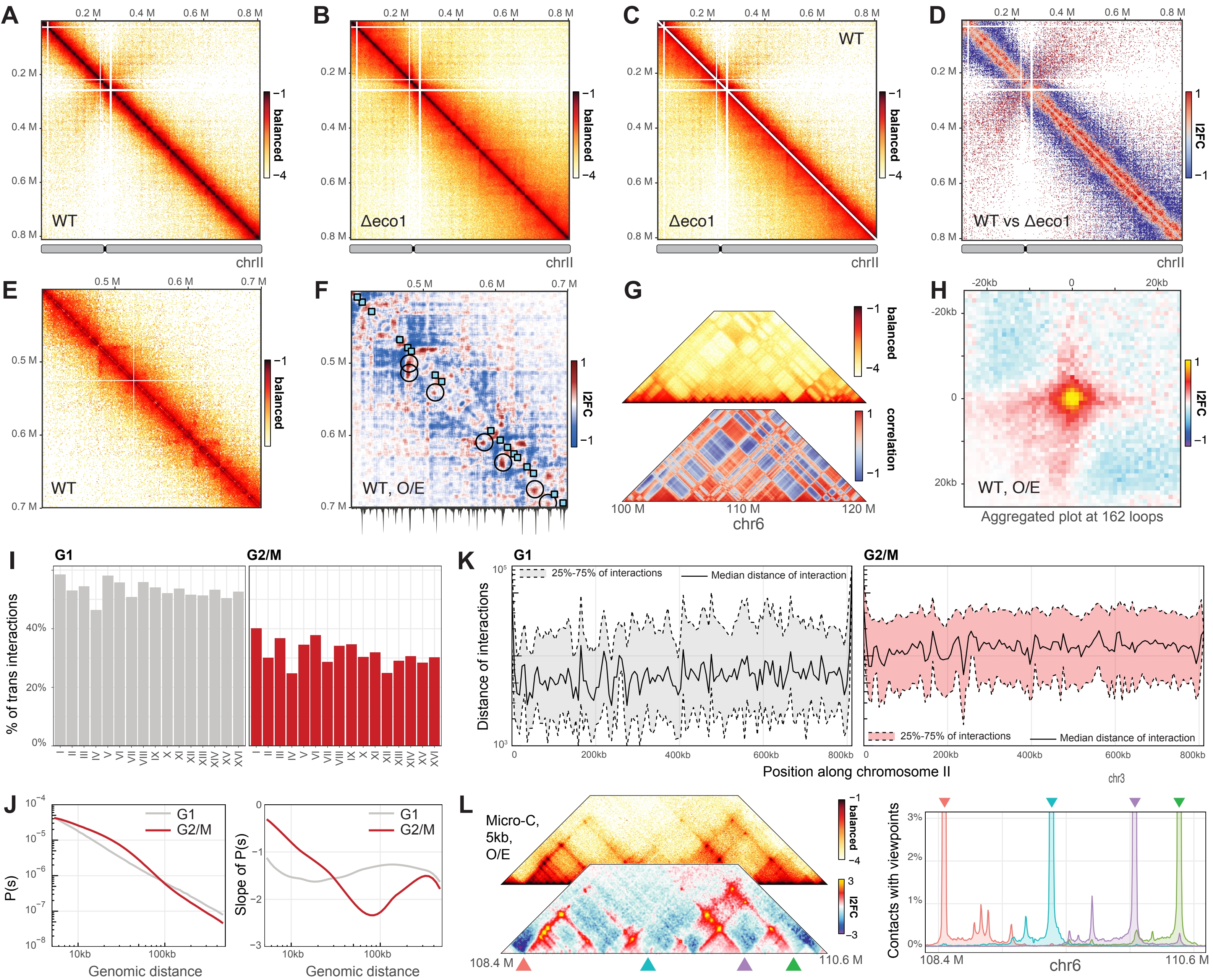

Serizay et al., Fig S2

Supplementary Figure 2: Visualization, matrix-centric and interaction-centric analysis of *HiCExperiment* objects

All the figures have been generated using *HiContacts* visualization tools. In some cases, legends have been subsequently manually rearranged.

A-B. Normalized Hi-C contact matrix of chrII for WT (A) or *eco1 mutant* (B) yeast (2kb bins).

C. Side-by-side comparison of normalized Hi-C contact matrices (chrII) of WT (top-right) and *eco1* mutant (bottom-left) yeast.

D. WT vs. *eco1* normalized Hi-C contact matrices ratio map (chrII). Red reflects interaction enrichment in WT whereas blue indicates interaction enrichment in *eco1* mutant.

E. Normalized Hi-C contact matrix of a 300kb segment of chrII for WT yeast (2kb bins).

F. Observed vs. expected (O/E) matrix of a 300kb segment of chrII for WT yeast (2kb bins). Red indicates more frequent interactions than expected by chance, whereas blue indicates less frequent interactions than expected by chance. Black circles highlight chromatin loops and blue diamonds denote domain borders, both identified by *chromosight*. The linear track below the Hi-C map represents aligned Scc1 (cohesin) ChIP-seq profile.

G. Normalized interaction frequency (top) and correlation matrix (bottom) of a micro-C dataset generated in HFFc6 cell line (Krietenstein et al., 2020), over a 20Mb segment of chr6. The upper triangular matrix representation is natively supported by *HiContacts*.

H. Aggregated yeast Hi-C map over 162 off-diagonal locations extracted from the genome-wide Hi-C dataset. The color code indicates the O/E signal averaged over the 162 snippets.

I. Inter-chromosomal interactions in WT yeast cells synchronized in G1 (left, grey) or G2/M (right, red) phases.

J. Distance-dependent interaction frequency (P(s)) and its slope in the two respective yeast Hi-C datasets.

K. Scalograms of interactions over chrII in the two respective yeast Hi-C datasets. The shaded area represents the span of genomic distances at which 50% of the interactions occur. Note how the median distance of interaction is increased across the entire chrII in the G2/M yeast dataset (red).

L. O/E interaction scores in a micro-C dataset generated in HFFc6 cell line (Krietenstein et al., 2020), over a 2.2Mb segment of chr6 (left) and profiles of interactions (virtual 4C profiles) of four viewpoints indicated by colored arrows (right).

	Function	Input class	Output class	Extra required arguments
Matrix-centric functions	normalize	HiCExperiment	HiCExperiment with added `ICE` score	n/a
	divide	2 HiCExperiments	1 HiCExperiment with divided scores	n/a
	merge	List of HiCExperiments	1 HiCExperiment with averaged/summed scores	n/a
	detrend	HiCExperiment	HiCExperiment with added `expected` and `detrended scores	n/a
	autocorrelate	HiCExperiment	HiCExperiment with added `autocorrelated` score	n/a
	despeckle	HiCExperiment	HiCExperiment with added `despeckled` score	n/a
	aggregate	HiCExperiment	AggrHiCExperiment with `slices` slot and `targets` topologicalFeatures	targets (GRanges or GInteractions)
	subsample	HiCExperiment	HiCExperiment with subsampled interactions	prop (float or integer)
	coarsen	HiCExperiment	HiCExperiment with coarser resolution	bin.size (integer)
Interaction-centric functions	distanceLaw	HiCExperiment	data.frame	n/a
	virtual4C	HiCExperiment	GRanges	viewpoint (GRanges)
	cisTransRatio	HiCExperiment	data.frame	n/a
	localDistanceLaw	HiCExperiment	data.frame	coords (GRanges)
	scalogram	HiCExperiment	data.frame	n/a
Feature mapping	getCompartments	HiCExperiment	HiCExperiment with added `compartments` topologicalFeatures and `eigen` metadata	n/a
	getDiamondInsulation	HiCExperiment	HiCExperiment with added `insulation` metadata	n/a
	getBorders	HiCExperiment	HiCExperiment with added `borders` topologicalFeatures	n/a
	getLoops	HiCExperiment	HiCExperiment with added `loops` topologicalFeatures and `chromosight_args` metadata	n/a
Visualization	plotMatrix	HiCExperiment, AggrHiCExperiment, Ginteractions, matrix	ggplot	n/a
	plotPs	HiCExperiment	ggplot	mapping (aes)
	plotPsSlope	HiCExperiment	ggplot	mapping (aes)
	plot4C	HiCExperiment	ggplot	n/a
	plotScalogram	HiCExperiment	ggplot	n/a
	plotSaddle	HiCExperiment	ggplot	n/a

Serizay et al., Table S1

Supplementary Table 1: Overview of *HiContacts* functions to visualize and investigate *HiCExperiment* objects

For each function, the input class, the output class and the other required arguments are listed.
