# Supplementary material for: Orchestrating chromosome conformation capture analysis with Bioconductor
Source: Nat Commun. 2024 Feb 5;15:1072. doi: 10.1038/s41467-024-44761-x (PMC10844600; doi:10.1038/s41467-024-44761-x)
Supplement: Supplementary file 3 — Reporting Summary [file 41467_2024_44761_MOESM3_ESM.pdf]

nature portfolio

Corresponding author(s): Jacques Serizay

Last updated by author(s): Dec 7, 2023

Reporting Summary

Nature Portfolio wishes to improve the reproducibility of the work that we publish. This form provides structure for consistency and transparency in reporting. For further information on Nature Portfolio policies, see our Editorial Policies and the Editorial Policy Checklist.

Statistics

For all statistical analyses, confirm that the following items are present in the figure legend, table legend, main text, or Methods section.

n/a	Confirmed	
☐	☒	The exact sample size (*n*) for each experimental group/condition, given as a discrete number and unit of measurement
☒	☐	A statement on whether measurements were taken from distinct samples or whether the same sample was measured repeatedly
☐	☒	The statistical test(s) used AND whether they are one- or two-sided<br>*Only common tests should be described solely by name; describe more complex techniques in the Methods section.*
☒	☐	A description of all covariates tested
☒	☐	A description of any assumptions or corrections, such as tests of normality and adjustment for multiple comparisons
☐	☒	A full description of the statistical parameters including central tendency (e.g. means) or other basic estimates (e.g. regression coefficient) AND variation (e.g. standard deviation) or associated estimates of uncertainty (e.g. confidence intervals)
☐	☒	For null hypothesis testing, the test statistic (e.g. *F*, *t*, *r*) with confidence intervals, effect sizes, degrees of freedom and *P* value noted<br>*Give P values as exact values whenever suitable.*
☒	☐	For Bayesian analysis, information on the choice of priors and Markov chain Monte Carlo settings
☒	☐	For hierarchical and complex designs, identification of the appropriate level for tests and full reporting of outcomes
☒	☐	Estimates of effect sizes (e.g. Cohen's *d*, Pearson's *r*), indicating how they were calculated

Our web collection on statistics for biologists contains articles on many of the points above.

Software and code

Policy information about availability of computer code

Data collection	Yeast Hi-C data come from (Dauban et al., 2020) and fastq files were obtained from the SRA repository (SRA accession numbers: SRR8769554, SRR10687276, SRR8769549, SRR10687281, SRR8769551, SRR10687278, SRR8769555) or directly obtained through HiContactsData. Yeast ChIP-seq data come from (Chapard et al., 2022) and processed data were obtained from GEO (GSM6703614). Chicken Hi-C data come from (Gibcus et al., 2018) and was directly imported from the 4DN data portal with fourDNData (ExperimentSet accession numbers: 4DNES9LEZXN7, 4DNESNWWIFZU, 4DNESGDXKM2I, 4DNESIR416OW, 4DNESS8PTK6F). micro-C data generated from HFFc6 cells (Krietenstein et al., 2020) was also imported from the 4DN data portal (ExperimentSet accession number: 4DNESWST3UBH).
Data analysis	All the analysis steps are extensively described as dedicated workflows in the companion website: https://js2264.github.io/OHCA/. Additional examples are also available from the following documentation webpages: Importing Hi-C data (https://js2264.github.io/HiCExperiment/reference/HiCExperiment.html#ref-examples), Arithmetic with Hi-C data (https://js2264.github.io/HiContacts/reference/arithmetics.html#examples) and Plotting Hi-C matrices (https://js2264.github.io/HiContacts/reference/plotMatrix.html). HiCExperiment is freely available on Bioconductor (https://bioconductor.org/packages/HiCExperiment), and the source code is hosted on a GitHub repository (https://github.com/js2264/HiCExperiment). HiContacts, HiCool, fourDNData and DNAZooData packages are also freely provided as Bioconductor packages (https://bioconductor.org/packages) and publicly hosted on GitHub. hicstuff is publicly available as a standalone python package from bioconda.<br><br>All packages were installed in R using Bioconductor 3.18.

For manuscripts utilizing custom algorithms or software that are central to the research but not yet described in published literature, software must be made available to editors and reviewers. We strongly encourage code deposition in a community repository (e.g. GitHub). See the Nature Portfolio guidelines for submitting code & software for further information.

Data

Policy information about availability of data

All manuscripts must include a data availability statement. This statement should provide the following information, where applicable:

- Accession codes, unique identifiers, or web links for publicly available datasets
- A description of any restrictions on data availability
- For clinical datasets or third party data, please ensure that the statement adheres to our policy

All data presented in this manuscript have already been published. Yeast Hi-C data come from (Dauban et al., 2020) and fastq files were obtained from the SRA repository (SRA accession numbers: SRR8769554, SRR10687276, SRR8769549, SRR10687281, SRR8769551, SRR10687278, SRR8769555) or directly obtained through HiContactsData. Yeast ChIP-seq data come from (Chapard et al., 2022) and processed data were obtained from GEO (GSM6703614). Chicken Hi-C data come from (Gibcus et al., 2018) and was directly imported from the 4DN data portal with fourDNData (ExperimentSet accession numbers: 4DNES9LEZXN7, 4DNESNWWIFZU, 4DNESGDXKM2I, 4DNESIR416OW, 4DNESS8PTK6F). micro-C data generated from HFFc6 cells (Krietenstein et al., 2020) was also imported from the 4DN data portal (ExperimentSet accession number: 4DNESWST3UBH).

Research involving human participants, their data, or biological material

Policy information about studies with human participants or human data. See also policy information about sex, gender (identity/presentation), and sexual orientation and race, ethnicity and racism.

Reporting on sex and gender	NA
Reporting on race, ethnicity, or other socially relevant groupings	NA
Population characteristics	NA
Recruitment	NA
Ethics oversight	NA

Note that full information on the approval of the study protocol must also be provided in the manuscript.

Field-specific reporting

Please select the one below that is the best fit for your research. If you are not sure, read the appropriate sections before making your selection.

☒ Life sciences ☐ Behavioural & social sciences ☐ Ecological, evolutionary & environmental sciences

For a reference copy of the document with all sections, see nature.com/documents/nr-reporting-summary-flat.pdf

Life sciences study design

All studies must disclose on these points even when the disclosure is negative.

Sample size	No sample size calculation was performed.
Data exclusions	No data was excluded.
Replication	Published replicate Hi-C experiments were obtained from SRA, GEO and the 4DN data portal when available.
Randomization	No randomization was required in this study.
Blinding	No blinding was required in this study.

Reporting for specific materials, systems and methods

We require information from authors about some types of materials, experimental systems and methods used in many studies. Here, indicate whether each material, system or method listed is relevant to your study. If you are not sure if a list item applies to your research, read the appropriate section before selecting a response.

Materials & experimental systems

n/a	Involved in the study
☒	☐ Antibodies
☒	☐ Eukaryotic cell lines
☒	☐ Palaeontology and archaeology
☒	☐ Animals and other organisms
☒	☐ Clinical data
☒	☐ Dual use research of concern
☒	☐ Plants

Methods

n/a	Involved in the study
☒	☐ ChIP-seq
☒	☐ Flow cytometry
☒	☐ MRI-based neuroimaging
